# Brain morphological analysis in mice with hyperactivation of the hedgehog signaling pathway

**DOI:** 10.3389/fnins.2024.1449673

**Published:** 2024-09-03

**Authors:** Tadashi Shiohama, Hideki Uchikawa, Nobuhiro Nitta, Tomozumi Takatani, Shingo Matsuda, Alpen Ortug, Emi Takahashi, Daisuke Sawada, Eiji Shimizu, Katsunori Fujii, Ichio Aoki, Hiromichi Hamada

**Affiliations:** ^1^Department of Pediatrics, Graduate School of Medicine, Chiba University, Chiba, Japan; ^2^Department of General Medical Science, Graduate School of Medicine, Chiba University, Chiba, Japan; ^3^Institute for Quantum Medical Science, National Institutes for Quantum Science and Technology, QST, Chiba, Japan; ^4^Central Institute for Experimental Medicine and Life Science Bio Imaging Center, Yokohama, Japan; ^5^Department of Cognitive Behavioral Physiology, Graduate School of Medicine, Chiba University, Chiba, Japan; ^6^Department of Pharmacotherapeutics, Showa Pharmaceutical University, Tokyo, Japan; ^7^Department of Ultrastructural Research, National Institute of Neuroscience, National Center of Neurology and Psychiatry, Tokyo, Japan; ^8^Athinoula A. Martinos Center for Biomedical Imaging, Massachusetts General Hospital, Boston, MA, United States; ^9^Department of Radiology, Harvard Medical School, Boston, MA, United States; ^10^Department of Pediatrics, International University of Health and Welfare Narita Hospital, Narita, Japan

**Keywords:** hedgehog signaling, *Ptch*, Gorlin syndrome, brain magnetic resonance imaging, laterality

## Abstract

Hedgehog signaling is a highly conserved pathway that plays pivotal roles in morphogenesis, tumorigenesis, osteogenesis, and wound healing. Previous investigations in patients with Gorlin syndrome found low harm avoidance traits, and increased volumes in the cerebrum, cerebellum, and cerebral ventricles, suggesting the association between brain morphology and the constitutive hyperactivation of hedgehog signaling, while the changes of regional brain volumes in upregulated hedgehog signaling pathway remains unclear so far. Herein, we investigated comprehensive brain regional volumes using quantitative structural brain MRI, and identified increased volumes of amygdala, striatum, and pallidum on the global segmentation, and increased volumes of the lateral and medial parts of the central nucleus of the amygdala on the detail segmentation in *Ptch* heterozygous deletion mice. Our data may enhance comprehension of the association between brain morphogenic changes and hyperactivity in hedgehog signaling.

## 1 Introduction

Hedgehog signaling is a highly conserved pathway that plays pivotal roles in morphogenesis, tumorigenesis, osteogenesis, and wound healing. The hedgehog signaling pathway is initiated by the binding of the hedgehog protein to its membrane receptor Patched (Ptch), resulting in the inhibition of the repression of the G protein-coupled receptor family protein Smoothened (Smo). SMO intracellularly activates hedgehog signaling through several cytoplasmic transduction steps, leading to the nuclear action of Gli proteins, which subsequently regulate target genes (Ruiz i Altaba et al., 2002; Andreu-Cervera et al., 2021). Especially, hedgehog protein is one of the most important morphogens that displays pleiotropic functions during embryonic development, ranging from neuronal patterning to axon guidance (reviewed by Douceau et al., 2023; Avilés et al., 2013).

Among the morphogenetic adjustments by hedgehog signaling, its contribution to fetal brain development is well recognized. Hedgehog signaling controls brain morphology mainly by regulating neuronal proliferation, forebrain development, and cerebellar development (Davies and Miller, 2001; Komada et al., 2008; Komada, 2012; Radonjić et al., 2016; Kiyasova and Gaspar, 2011; Abeliovich and Hammond, 2007; Fernandes and Hébert, 2008; Solomon et al., 2012; Andreu-Cervera et al., 2021; Vaillant and Monard, 2009; Corrales et al., 2004; Shiohama et al., 2017; Wechsler-Reya and Scott, 1999). Hedgehog signaling also positively regulates the proliferation of neural stem cells and oligodendroglia in the neocortex (Davies and Miller, 2001; Komada et al., 2008; Komada, 2012; Radonjić et al., 2016), serotonin-producing neurons (Kiyasova and Gaspar, 2011), and dopaminergic neurons (Abeliovich and Hammond, 2007). Hedgehog signaling is also essential for the formation of the midline structure of the forebrain, and defects in this signaling pathway have been shown to lead to holoprosencephaly (Fernandes and Hébert, 2008; Solomon et al., 2012; Andreu-Cervera et al., 2021). In addition, Hedgehog signaling positively controls cerebellar size in both mice (Vaillant and Monard, 2009; Corrales et al., 2004) and humans (Shiohama et al., 2017) by driving the proliferation of cerebellar granular cells (Wechsler-Reya and Scott, 1999).

Personality psychology has attracted considerable attention in personality disorders (Stockings et al., 2016), childhood adversities such as bullying and child abuse (Peh et al., 2019), bipolar disorders (Luciano et al., 2021), and Parkinson’s disorders (Santangelo et al., 2018). To assess personality, Cloninger’s biosocial model of personality assesses the four dimensions of fundamental temperament: novelty seeking, harm avoidance, reward dependence, and persistence, which have been widely used in children and adults (Cloninger et al., 1993; Hansenne and Ansseau, 1999; Däschle et al., 2023). Each temperament is independently heritable and associated with neurotransmitters. Novelty seeking is associated with dopaminergic activity, harm avoidance is associated with serotonergic activity, reward dependence is associated with noradrenergic activity, and persistence is associated with unknown neurotransmitters (Cloninger, 1987; Hansenne and Ansseau, 1999).

Harm avoidance is a personality trait characterized by excessive worry, fear of uncertainty, shyness, and fatigue (Bey et al., 2017). Harm avoidance has strong heritability (Garcia et al., 2013; Keller et al., 2005), high stability throughout life (Josefsson et al., 2013), and tight connectivity with obsessive-compulsive disorder (Ettelt et al., 2008; Bey et al., 2017), depression, and anxiety (Kenna et al., 2012; Meylakh and Henderson, 2016). However, the relationship among harm avoidance traits, brain morphology, and neurotransmitters remains unclear.

Magnetic resonance imaging (MRI) is a well-established tool in clinical practice and research on disorders with various conditions including neurodevelopmental disorders, neurodegenerative disorders, and psychiatric disorders/personality traits. The importance of neuroimaging in rodents has increased significantly and plays an important role in neuroscience research, translational medicine, and pharmaceutical advances (Liu et al., 2020). MRI studies in animal models (preclinical) aim to explore new aspects of disease processes that have not yet been fully understood in human diseases. Animal models are crucial for the scientific study of the normal physiological mechanisms that regulate both normal and abnormal behavior, as well as pathological outliers and processes (van der Staay et al., 2009). Many mouse models have been used to study inflammatory diseases of the central nervous system, neurodegenerative diseases, stroke, brain and spinal cord trauma models, as well as brain tumors, through MRI (Denic et al., 2011). However, to the best of our knowledge, this is the first study to correlate harm avoidance and Hedgehog signaling in a mouse model using a preclinical structural MRI technique.

Our previous study revealed a characteristic personal pattern with low levels of harm avoidance in patients with Gorlin syndrome (OMIM #109400) due to the *PTCH1* pathogenic variant, suggesting that congenital hyperactivity in hedgehog signaling may contribute to controlling harm avoidance on biosocial characteristics (Uchikawa et al., 2021). In this study, we investigated the brain morphological changes by the hyperactivity in hedgehog signaling using high-resolution structural MRI studies in *Ptch* heterozygous deletion mice.

## 2 Materials and methods

### 2.1 Experimental animals

All animal care and treatments were performed in accordance with the guidelines of the Experimental Animal Care Committee of Chiba University. The PTCH+/− mice (B6.Cg-Ret<tm1Mat> PTCH1 < tm1Mps>) (Goodrich et al., 1997) were provided by the RIKEN BRC through the National Bio-Resource Project of the MEXT, Japan. This genetically engineered mouse with *Ptch* heterozygous knockout has long been recognized as a mouse with enhanced hedgehog signaling (Goodrich et al., 1997; Aszterbaum et al., 1999; Rigby et al., 2019). All mice used in this study, both mutant (PTCH +/−) and wildtype (WT) littermates, were bred from wildtype C57BL/6 J females and Ptch1<tm1Mps> heterozygous males. All mice were housed 2–5 per cage and maintained on a 12-h light/dark cycle with water and food available *ad libitum*.

### 2.2 Genotyping

After weaning, mouse genomic DNA was extracted from the tip of the tail using phenol-chloroform DNA extraction, and a polymerase chain reaction strategy was applied to distinguish WT from mutant alleles using the GoTaq Green Master Mix (#M7122, Promega, Madison, WI, United States) following standard methodologies. The cycling conditions were 94°C for 2 min, followed by 32 cycles of 94°C for 30 s, 59°C for 0 s, and 72°C for 90 s. After 32 PCR cycles, the procedure was examined by electrophoresis on a 2% agarose gel. Run on 2% agarose gel. Wild-type (forward primer, TGG GGT GGG ATT AGA TAA ATG CC; reverse primer, TGT CTG TGT GTG CTC CTG AAT CAC) and mutant bands (forward primer, CTG CGG CAA GTT TTT GGT TG; reverse primer, AGG GCT TCT CGT TGG CTA CAAG) were identified at 217 and 501 bp, respectively.

### 2.3 MRI acquisition and processing

#### 2.3.1 Animal procedure

Six 12-week-old male PTCH+/− mice and six 12-week-old male WT male mice were anesthetized by intraperitoneal injection of a mixture of medetomidine, midazolam, and butorphanol (Kirihara et al., 2013), and were sacrificed by transcardial perfusion with phosphate buffered saline and 4% paraformaldehyde. Mouse heads were decapitated and stored in 4% paraformaldehyde at 4°C until MRI scanning. The animal experimentation was conducted according to the protocol reviewed and approved by the institutional animal care and use committee of Chiba University (Permit No. 20–120).

#### 2.3.2 MRI acquisition

Mouse brains were scanned using a 7-tesla preclinical MRI scanner (Bruker BioSpin, Ettlingen, Germany), equipped with actively shielded gradients (BGA12S, 116 mm i.d., BrukerBioSpin) and a transmitting/receiving volume coil with an inner diameter of 22 mm. High resolution anatomical images of the whole brain were acquired using a Rapid Acquisition with Relaxation Enhancement (RARE) sequence with the following parameters: effective echo time (eTE) = 26 ms, repetition time (TR) = 2,500 ms, RARE factor = 4, number of averages = 4, spatial resolution = 70 × 70 × 70 μm3, scan time = 14 h 17 min 32 s.

#### 2.3.3 Automatic segmentation

The acquired structural T2-weighted images were analyzed using the Atlas Normalization Toolbox with elastiX version 2 (ANTx2) (Lein et al., 2007; Hübner et al., 2017; Koch et al., 2019)^1^ running in MATLAB (MathWorks, Natick, MA) toolbox for image registration of mouse MRI data. Through the ANTx2 pipeline, MR images were processed using SPM12^2^ and nonlinear warping of tissue probability maps in ELASTIX (Klein et al., 2010),^3^ and registered in the Allen Mouse Atlas 2017 (CCFv3) (Lein et al., 2007; Hikishima et al., 2017; Hübner et al., 2017). After checking the visual inspection of atlas registration, the estimated volumes of each anatomical region in the native space were individually calculated for each mouse. As global segmentation common mice to human, cerebrum, amygdala, striatum, pallidum, thalamus, hypothalamus, midbrain, pons, medulla, and cerebellum were selected for identifying candidate regions of volume change in PTCH +/− mice.

#### 2.3.4 Laterality index (LI)

To evaluate the structural asymmetry of regional brain volumes, we employed the LI (Springer et al., 1999), which was calculated as the ratio [VL—VR] / [VL + VR] × 100 (VL, volume of the left hemisphere; VR, volume of the right hemisphere). LIs were subsequently classified as left hemisphere dominant (defined as LI > 20), symmetric (−20 ≤ LI ≤ +20) or right hemisphere dominant (LI < −20).

### 2.4 Statistical analysis

GraphPad Prism version 9.5.1 (GraphPad Software, Boston, MA, United States) and Microsoft Excel 2019 (Redmond, WA, United States) were used for statistically analysis. The concentration of monoamines, brain regional weight, and brain measurements including the laterality index in 10 major segmentations were evaluated by Welch’s two-tailed unpaired *t*-tests (*p* < 0.05). Regional brain volumes were comprehensively evaluated through repeated Welch’s two-tailed unpaired t-tests with Benjamini-Hochberg methods (Benjamini et al., 2001; Reiner et al., 2003) for controlling the false discovery rate (FDR) (*q* = 0.1), rates of mean, and the absolute value of Cohen’s d statistic. Cohen’s d = 0.8 was recognized as the cut-off value for large-size effects (Cohen, 1992).

## 3 Results

### 3.1 Quantitative analysis of the brain morphology

**Figure 1:**
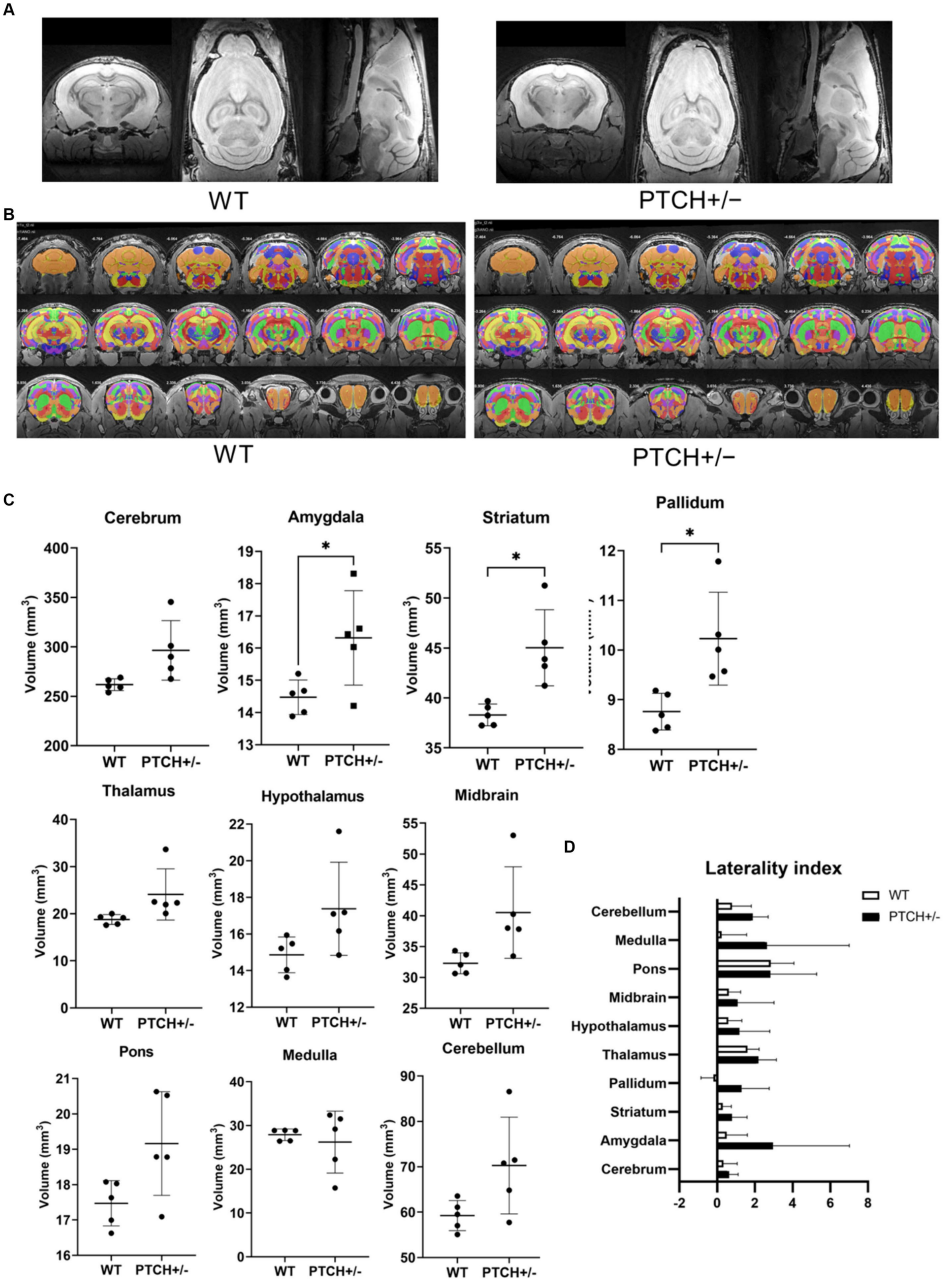
Brain morphology analysis using MRI. T2 weighted images **(A)**, atlas registration **(B)**, quantification of anatomical regions using volume-based morphometry **(C)**, and laterality index **(D)** in wild type (WT) and *Ptch* heterozygous deletion (PTCH+/−) mice. * indicates significantly difference (*p* < 0.05).

The six 12-week-old WT and PTCH+/− male mice were finally used for the brain morphologic study after exclusion of one PTCH+/− mouse with a medulloblastoma-like tumor in the cerebellum identified on brain MRI. Brain regional segmentation was performed in both PTCH+/− and WT mice, and measurements of 958 among 1,327 regions according to CCFv3 atlas were successfully determined for each mouse (Figures 1A,B, Supplementary Figure S1). Comparison of global regional volume showed statistically significant differences in the volumes of the amygdala, striatum, and pallidum between PTCH+/− and WT mice (Figure 1C). Although not reaching statistical significance, the volume of the cerebrum, thalamus, hypothalamus, midbrain, pons, and cerebellum tended to be higher in PTCH+/− mice than in WT mice.

The LI demonstrated a mild left hemisphere dominance in all parts of the global brain regions both in PTCH+/− mice and in WT mice (Figure 1D). No statistically significant difference of LI was identified between PTCH+/− and WT mice.

We subsequently compared the more detailed regional volumes of the amygdala, striatum, and pallidum of PTCH+/− and WT mice. Among the 11 regions of the amygdala, the lateral, basolateral, basomedial, posterior, striatum-like, central, intercalated, and medial part of amygdala nucleus showed statistically significant higher volume in PTCH+/− mice than in WT mice (Table 1). All of five parts of the striatum showed significantly higher volumes in PTCH+/− mice than in WT mice (Table 1). All of four parts of the pallidum showed statistically significant higher volume in PTCH+/− mice than in WT mice (Table 1).

**Table 1:** Comparison between brain region volumes of the amygdala, striatum, and pallidum of WT and PTCH +/− mice obtained using detailed volume-based morphometry.

Regions	WT mice (*N* = 5) mean [SD]	PTCH+/− mice (*N* = 5) mean [SD]	Rate of mean value of PTCH+/− to WT	Cohen’s D	Welch’s *t*-test *p* values
Amygdala total	14.5 [0.54]	16.3 [1.47]	1.12	1.63	0.046*
Cortical amygdala area	2.62 [0.13]	2.84 [0.28]	1.08	1.01	0.160
Piriform-amygdala area	0.77 [0.05]	0.79 [0.06]	1.03	0.36	0.550
Lateral amygdala nucleus	0.62 [0.03]	0.71 [0.09]	1.15	1.34	0.069*
Basolateral amygdala nucleus	1.45 [0.08]	1.61 [0.15]	1.11	1.33	0.085*
Basomedial amygdala nucleus	1.06 [0.06]	1.21 [0.13]	1.14	1.48	0.063*
Posterior amygdala nucleus	0.31 [0.02]	0.37 [0.04]	1.19	1.90	0.022*
Striatum-like amygdala nucleus	4.04 [0.14]	4.64 [0.43]	1.15	1.88	0.033*
Anterior amygdala area	0.60 [0.03]	0.66 [0.07]	1.10	1.11	0.120
Central amygdala nucleus	1.24 [0.05]	1.41 [0.13]	1.14	1.73	0.034*
Intercalated amygdala nucleus	0.22 [0.01]	0.25 [0.03]	1.14	1.34	0.060*
Medial amygdala nucleus	1.55 [0.05]	1.81 [0.18]	1.17	1.97	0.032*
Striatum total	38.3 [1.08]	45.02 [3.81]	1.18	2.40	0.015*
Striatum dorsal region	22.6 [0.59]	26.76 [2.29]	1.18	2.49	0.013*
Striatum ventral region	8.60 [0.39]	9.61 [0.64]	1.12	1.91	0.020*
Nucleus accumbens	4.39 [0.13]	4.91 [0.36]	1.12	1.92	0.026*
Fundus of striatum	0.40 [0.01]	0.45 [0.04]	1.13	1.72	0.047*
Olfactory tubercle	3.81 [0.26]	4.25 [0.26]	1.12	1.69	0.026*
Pallidum total	8.76 [0.37]	10.23 [0.93]	1.17	2.08	0.021*
Pallidum dorsal region	2.04 [0.15]	2.48 [0.23]	1.22	2.27	0.009*
Pallidum ventral region	3.52 [0.17]	3.94 [0.26]	1.12	1.91	0.021*
Pallidum medial region	1.80 [0.08]	2.13 [0.23]	1.18	1.92	0.029*
Pallidum caudal region	1.40 [0.05]	1.67 [0.26]	1.19	1.44	0.078*

*Denotes a *p*-value of less than 0.087 for 23 repeated t-test with false discovery rate correction (*q* = 0.1), which is statistically significant. Abbreviation; PTCH+/−, *Ptch* heterozygous deletion; SD, standard deviation; WT, wild type. We provided a supplemental statistical summary table about raw values, *p*-values of Welch’s *t*-test, FDR adjustment (*q* = 0.1), fold change, and Cohen’s D for all measurements in the neuroimaging study as Supplementary Table S1. These alterations were considered candidate at *p* < 0.005 and Cohen’s *D* > 0.8, but there are no brain regions with statistically significantly difference after adjustments for the multiple comparisons using FDR for 958 repeated *t*-test.

## 4 Discussion

In this study, we investigated comprehensive brain regional volumes using quantitative structural brain MRI, and identified increased volumes of amygdala, striatum, and pallidum on the global segmentation. There was a trend toward greater volumes of the infralimbic cortex and the lateral and medial parts of the central nucleus of the amygdala on the detail segmentation in *Ptch* heterozygous deletion mice. We chose *ex vivo* MRI instead of *in vivo* MRI to strictly match the scan week age, because of difficulty for preparing transgenic mice of the same sex and age siblings. Additionally, we scanned brain images over 14 h per mouse to visualize brain structure in detail and reduce signal noise ratio. *Ex vivo* MRI is not easily affected by motion artifacts, and susceptibility artifacts can be reduced by proper and careful sample preparation, such as avoiding bubble formation (Vasung et al., 2019).

In the current study, PTCH+/− mice showed increased volumes of the the lateral and medial parts of the central nucleus of the amygdala. The LI of the amygdala in PTCH+/− mice showed left hemispheric dominancy, although this did not reach statistical significance. The left amygdala has been identified as a region related to the level of harm avoidance through human resting-state functional MRI (Meylakh and Henderson, 2016), while in a semiquantitative brain MRI study on patients with Gorlin syndrome showed a smaller amygdala only on the left side (Uchikawa et al., 2021). The observations of the effect of increased Hedgehog signaling on amygdala volume differed between patients and model mice. Nees et al. (2020) found that in chronic pain patients, amygdala volume was not associated with pain avoidance itself but was significantly positively correlated with behavior to positive stimuli, which suggest that the amygdala’s response and volume changes related to harm avoidance may be more complex than in other brain regions.

Subsequently, we identified several morphological differences in the brain, using MRI, which may be associated with impaired harm avoidance. Harm avoidance is an adaptive defensive reaction to fear and anxiety (Robinson et al., 2019). Studies on the neural circuits of fear and anxiety (Calhoon and Tye, 2015; Robinson et al., 2019) have previously described that fear output is mediated by parts of the amygdala (the basolateral amygdala and the lateral and medial parts of the central nucleus of the amygdala) as well as the medial prefrontal cortex, and discussed that they are associated to harm avoidance. One functional MRI study also reported that the dorsal raphe nucleus, anterior cingulate cortex, and amygdala were correlated with harm avoidance (Meylakh and Henderson, 2016).

The lower harm avoidance was observed in our previous findings regarding the personality analysis in patients with Gorlin syndrome (Uchikawa et al., 2021). In the behavioral study of PTCH+/− mice, the open-field test showed early habituation, while the elevated plus maze test showed decreased anxiety-related behavior (Antonelli et al., 2018). In contrast to PTCH +/− mice, SMO-deficient mice, in which SHH signaling is suppressed, exhibit increased anxiety/depression-like behaviors without affecting spatial and fear-related learning ability (Wang et al., 2022). These findings support the hypothesis that hyperactive hedgehog signaling suppresses harm avoidance (Antonelli et al., 2018), and the extinction of fear memory is regulated by sonic hedgehog signaling (Hung et al., 2015). Harm avoidance is connected to the anxiety-related personality dimension (Meylakh and Henderson, 2016), and high harm avoidance scores are associated with anxiety and depression (Carver and Miller, 2006). In contrast, low harm avoidance scores are associated with risk-taking, harmful behavior, impulsiveness, suicidal ideation, and aggression (Peirson et al., 1999). Therefore, controlling the degree of harm avoidance could potentially aid in the development of novel therapies for psychological disorders.

The current study had some limitations. First, we evaluated brain morphology using MRI, but we did not employ a multimodal neuroimaging approach such as Blood Oxygenation Level Dependent (BOLD)-based functional MRI, perfusion/diffusion MRI, or PET/SPECT imaging. Although brain regional volume is widely recognized as a factor related to regional brain function, multimodal neuroimaging approaches could improve our understanding of the association between the neuroNetwork of hedgehog signaling activity. Second, it remains unclear whether the hedgehog signaling enhances is associated to harm avoidance. Further studies are therefore required to determine whether the activity level of hedgehog signaling. Third, our study was carried on only male mice to match sex, because the influence of sex differences on brain morphology cannot be ignored. Although the *Ptch* gene is not a gene on the sex chromosome, we cannot rule out the possibility that the results may be slightly altered in female mice. Differences between species may be another limitation of the present study. The comparison of human and mouse homolog cell types in the temporal lobe using single nucleus RNA-sequencing identified different patterns of gene expression in serotonin receptors, despite general conservation (Hodge et al., 2019); therefore, further investigation would be required to reveal whether the finding of the neuroimaging study could have much in common with humans.

In conclusion, we investigated comprehensive brain regional volumes using quantitative structural brain MRI, and identified increased volumes of the infralimbic cortex and the lateral and medial parts of the central nucleus of the amygdala in *Ptch* heterozygous deletion mice. Our data suggest that morphogenic changes in the neural circuits of harm avoidance may be connected to low harm avoidance and hyperactivity of hedgehog signaling.

## Data Availability

The original contributions presented in the study are included in the article/Supplementary material, further inquiries can be directed to the corresponding author.

## Footnotes

^1^ https://github.com/ChariteExpMri/antx2

^2^ https://www.fil.ion.ucl.ac.uk/spm/software/spm12

^3^ https://elastix.lumc.nl
