# Supplementary material for: Brain morphological analysis in mice with hyperactivation of the hedgehog signaling pathway
Source: Front Neurosci. 2024 Sep 3;18:1449673. doi: 10.3389/fnins.2024.1449673 (PMC11405378; doi:10.3389/fnins.2024.1449673)
Supplement: Supplementary file 2 [file Image_1.pdf]

Supplementary figure 1

WT_1

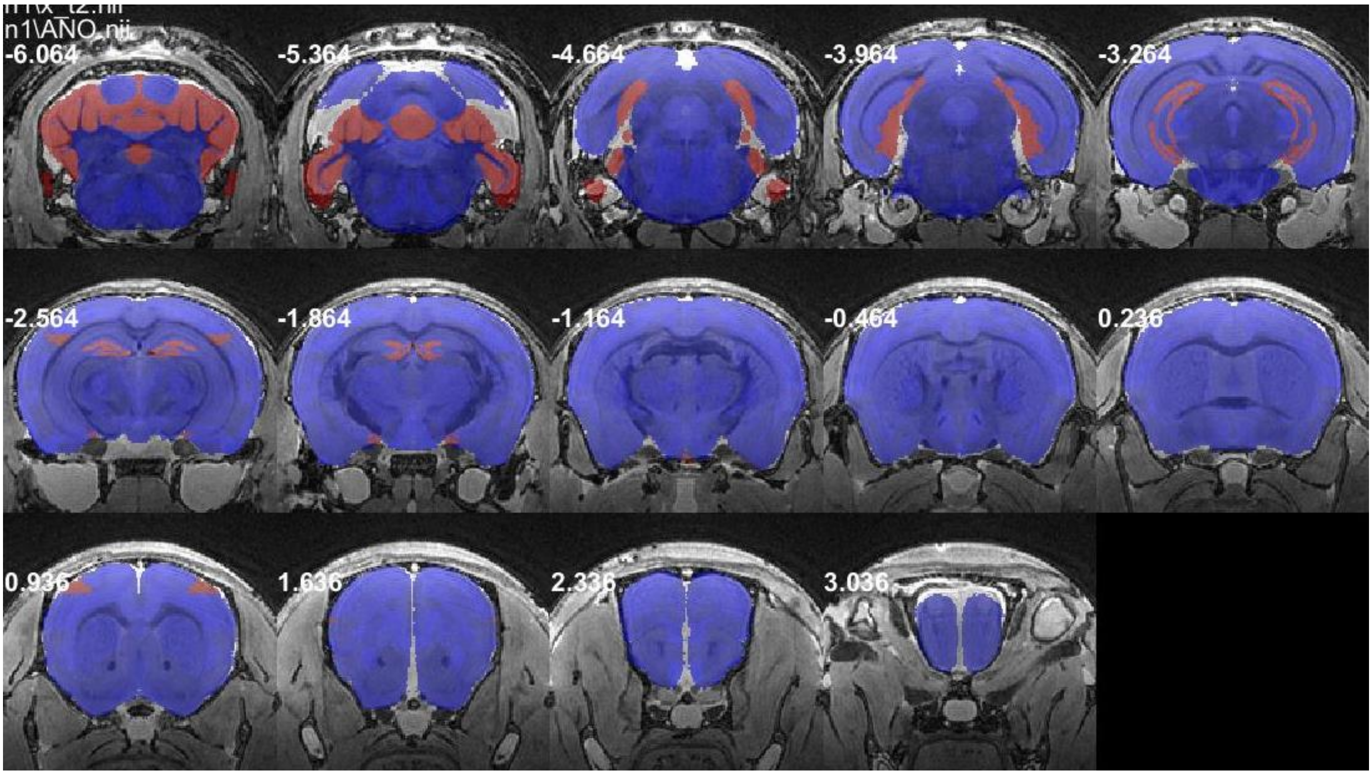

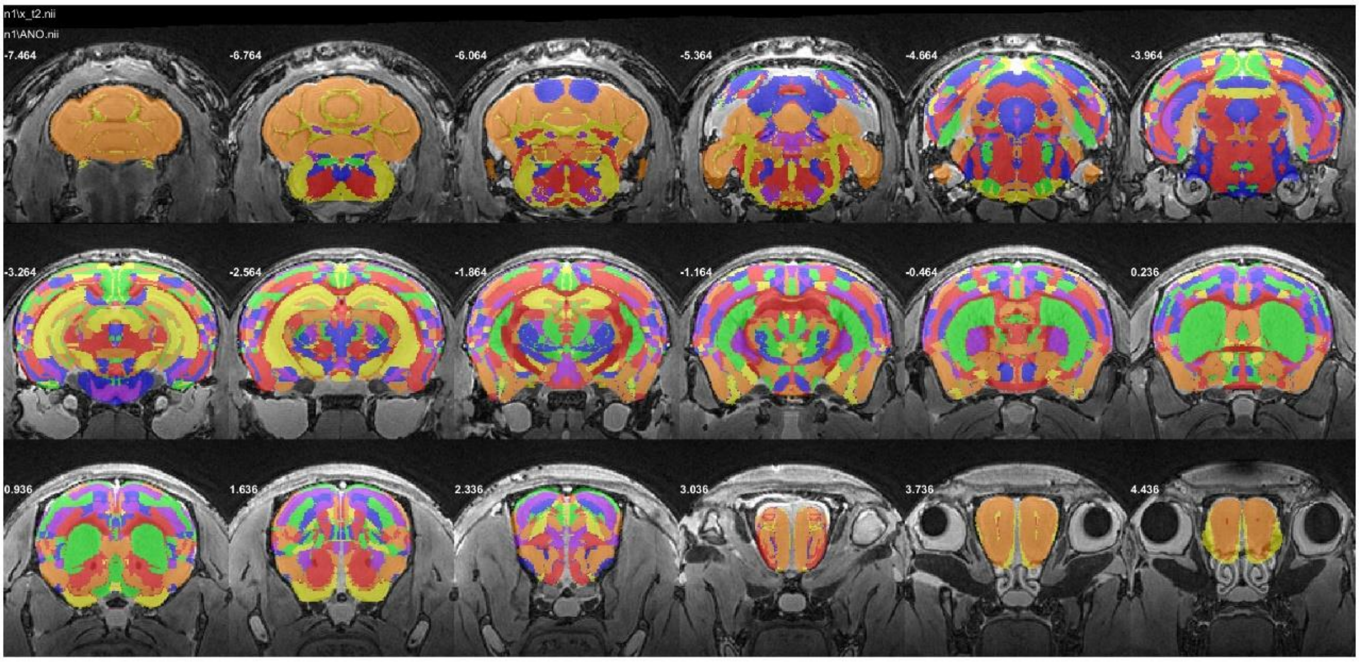

WT_2

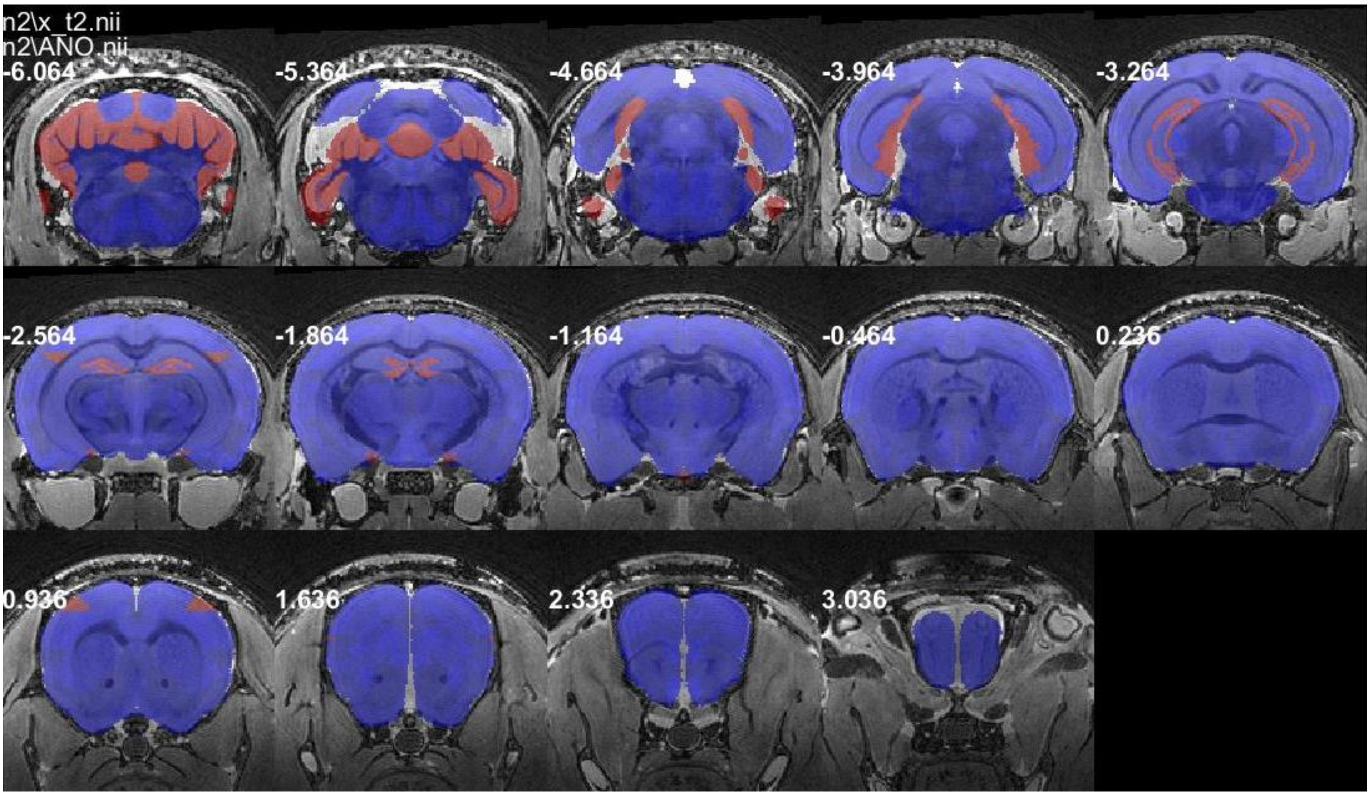
n2\x_t2.nii
n2\ANO.nii
-6.064
-5.364
-4.664
-3.964
-3.264
-2.564
-1.864
-1.164
-0.464
0.236
0.936
1.636
2.336
3.036

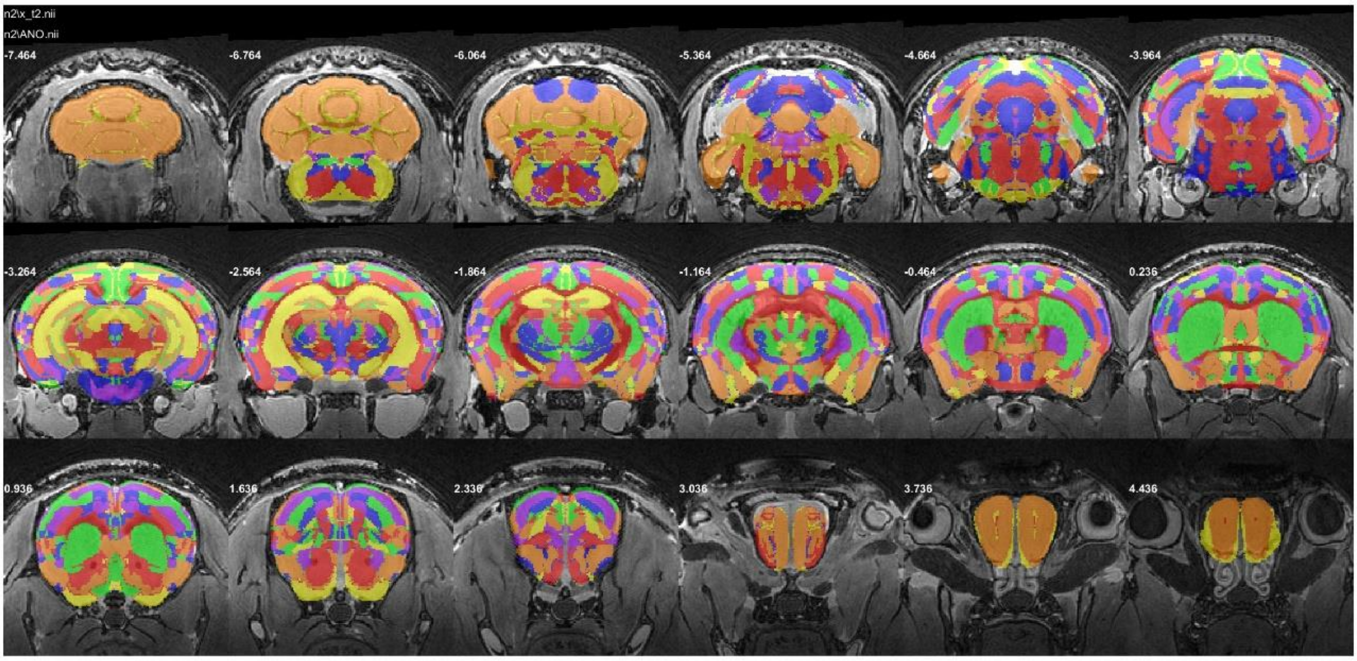
n2\x_t2.nii
n2\ANO.nii
-7.464
-6.764
-6.064
-5.364
-4.664
-3.964
-3.264
-2.564
-1.864
-1.164
-0.464
0.236
0.936
1.636
2.336
3.036
3.736
4.436

WT_3

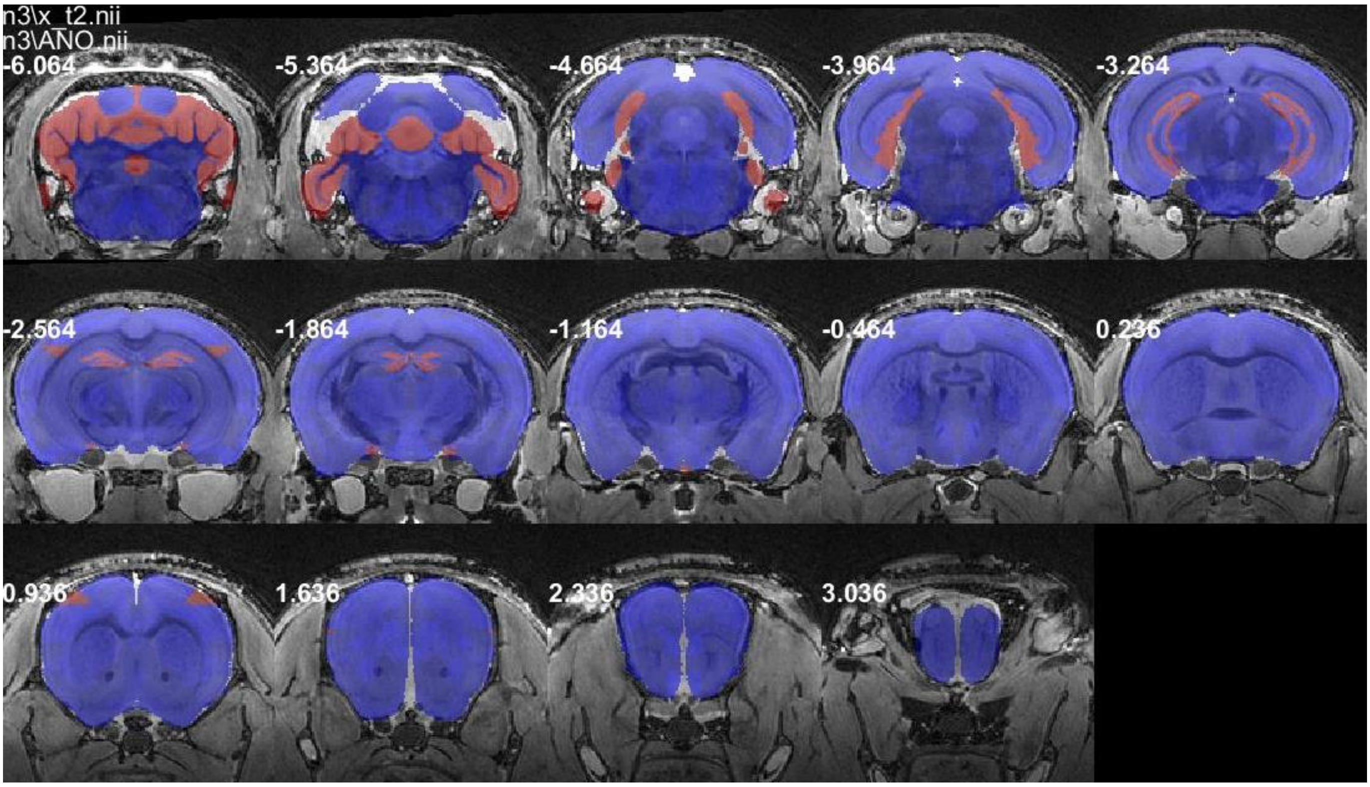
n3\x_t2.nii
n3\ANO.nii
-6.064
-5.364
-4.664
-3.964
-3.264
-2.564
-1.864
-1.164
-0.464
0.236
0.936
1.636
2.336
3.036

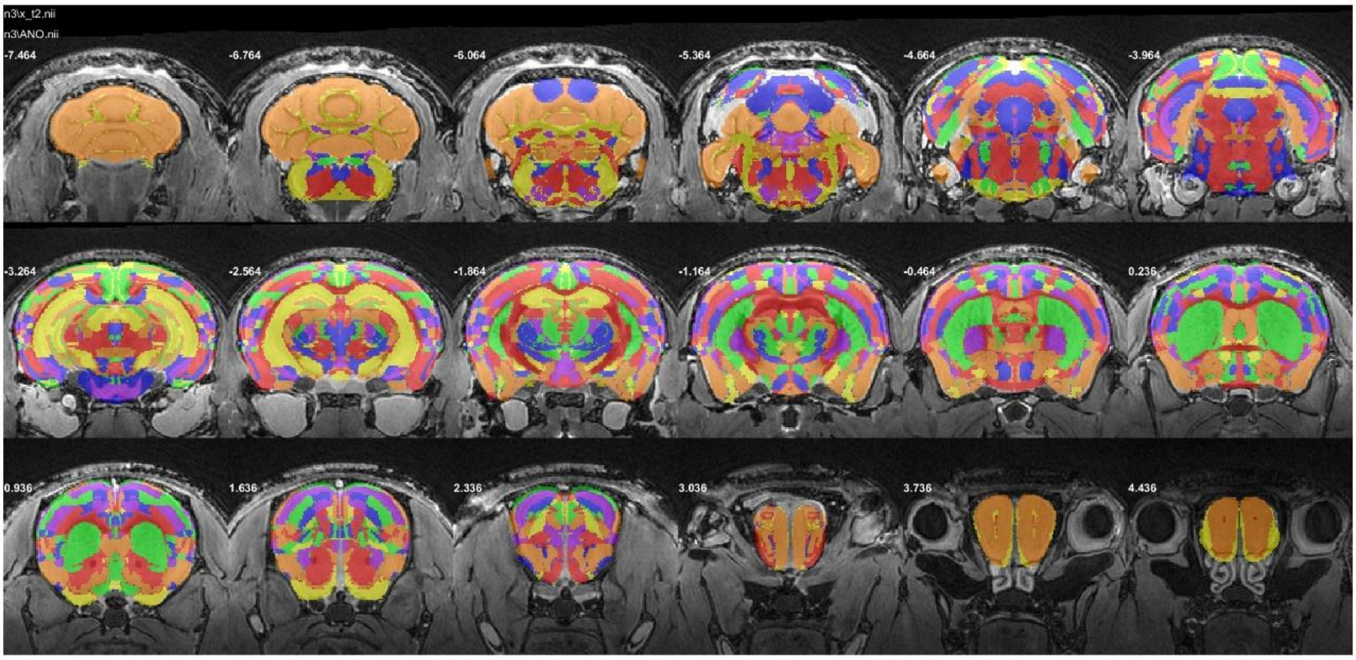
n3\x_t2.nii
n3\ANO.nii
-7.464
-6.764
-6.064
-5.364
-4.664
-3.964
-3.264
-2.564
-1.864
-1.164
-0.464
0.236
0.936
1.636
2.336
3.036
3.736
4.436

WT_4

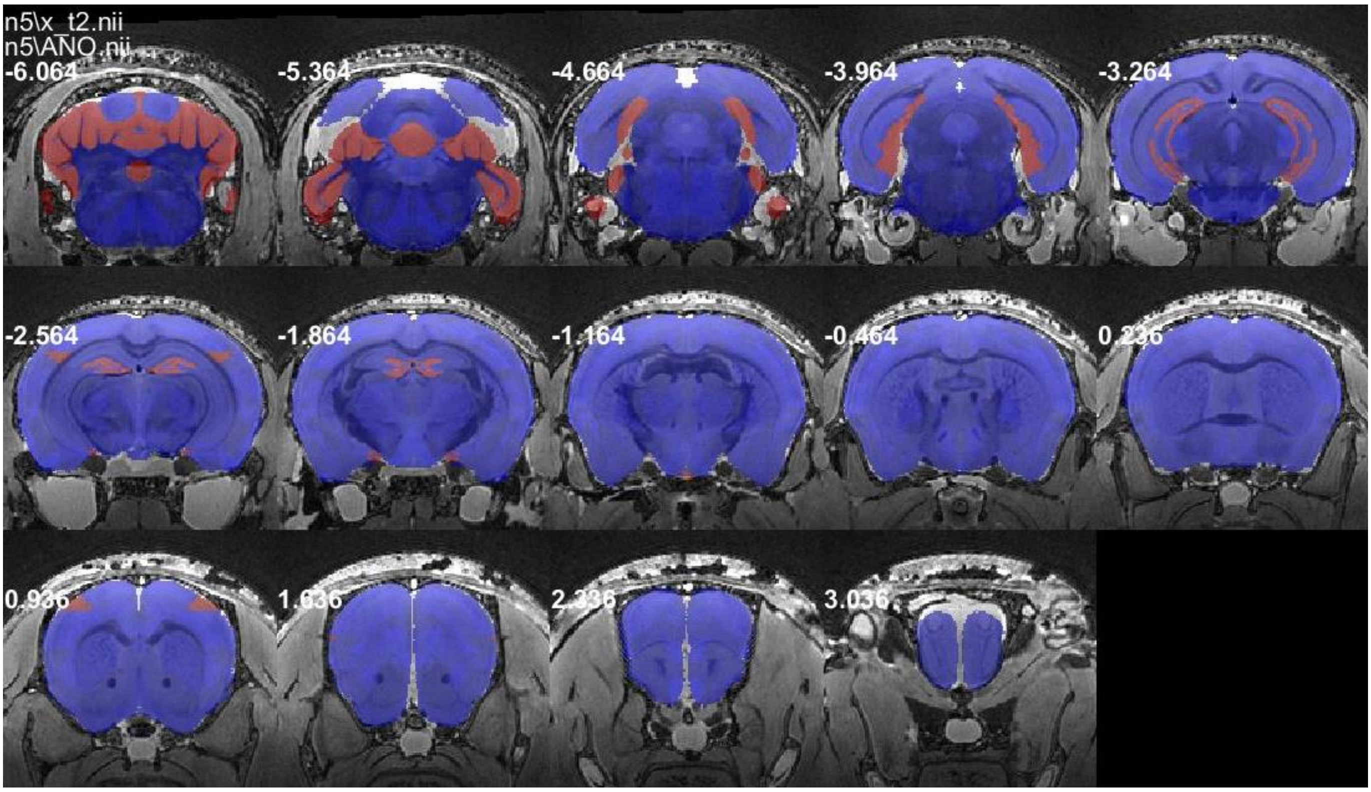
n5\x_t2.nii
n5\ANO.nii
-6.064
-5.364
-4.664
-3.964
-3.264
-2.564
-1.864
-1.164
-0.464
0.236
0.936
1.636
2.336
3.036

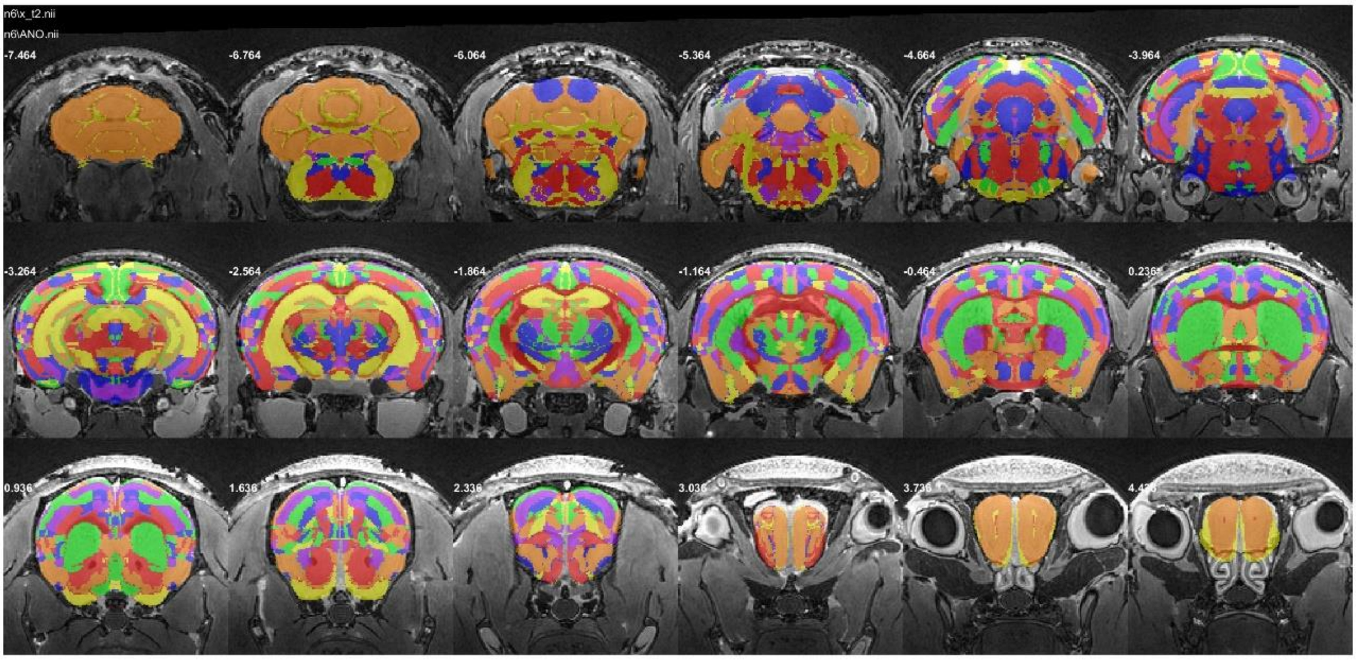
n6\x_t2.nii
n6\ANO.nii
-7.464
-6.764
-6.064
-5.364
-4.664
-3.964
-3.264
-2.564
-1.864
-1.164
-0.464
0.236
0.936
1.636
2.336
3.036
3.736

WT_5

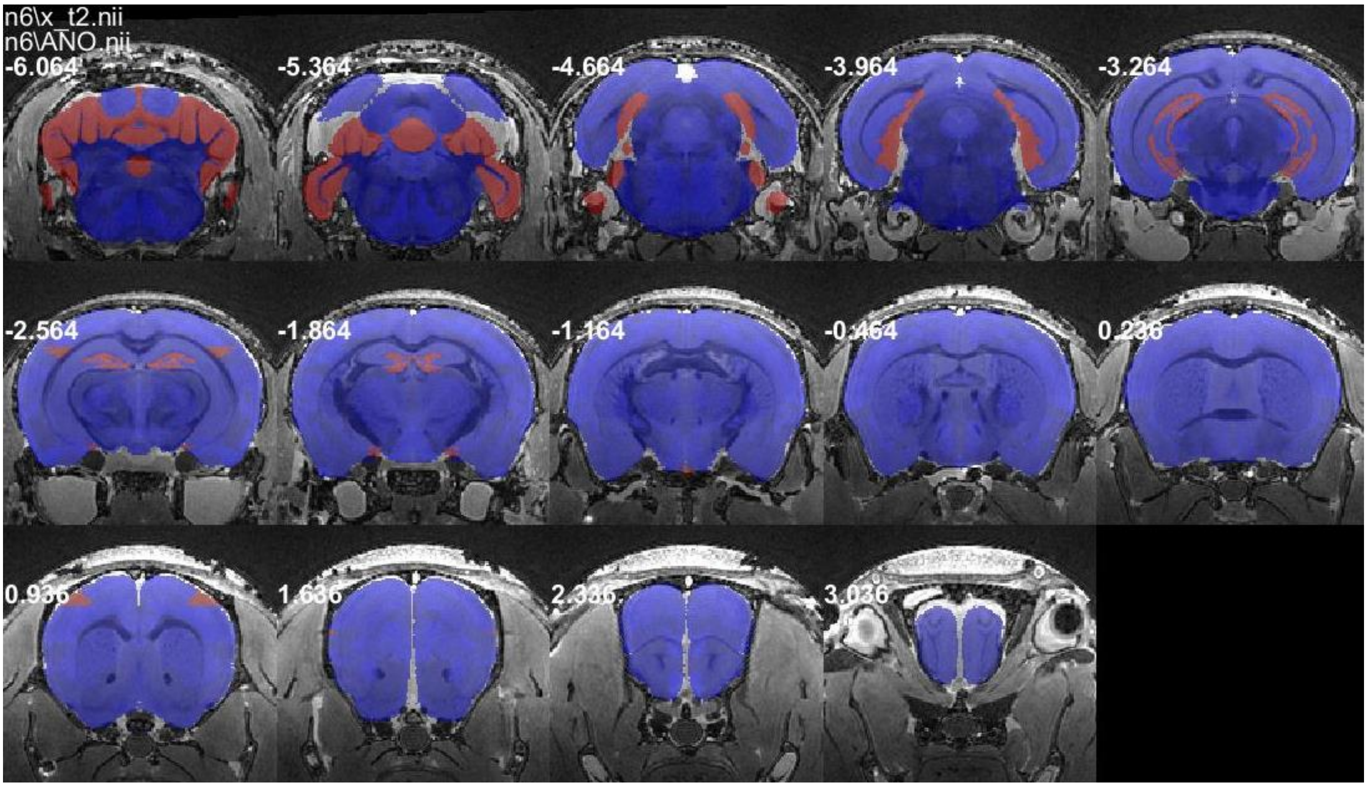
n6\x_t2.nii
n6\ANO.nii
-6.064
-5.364
-4.664
-3.964
-3.264
-2.564
-1.864
-1.164
-0.464
0.236
0.936
1.636
2.336
3.036

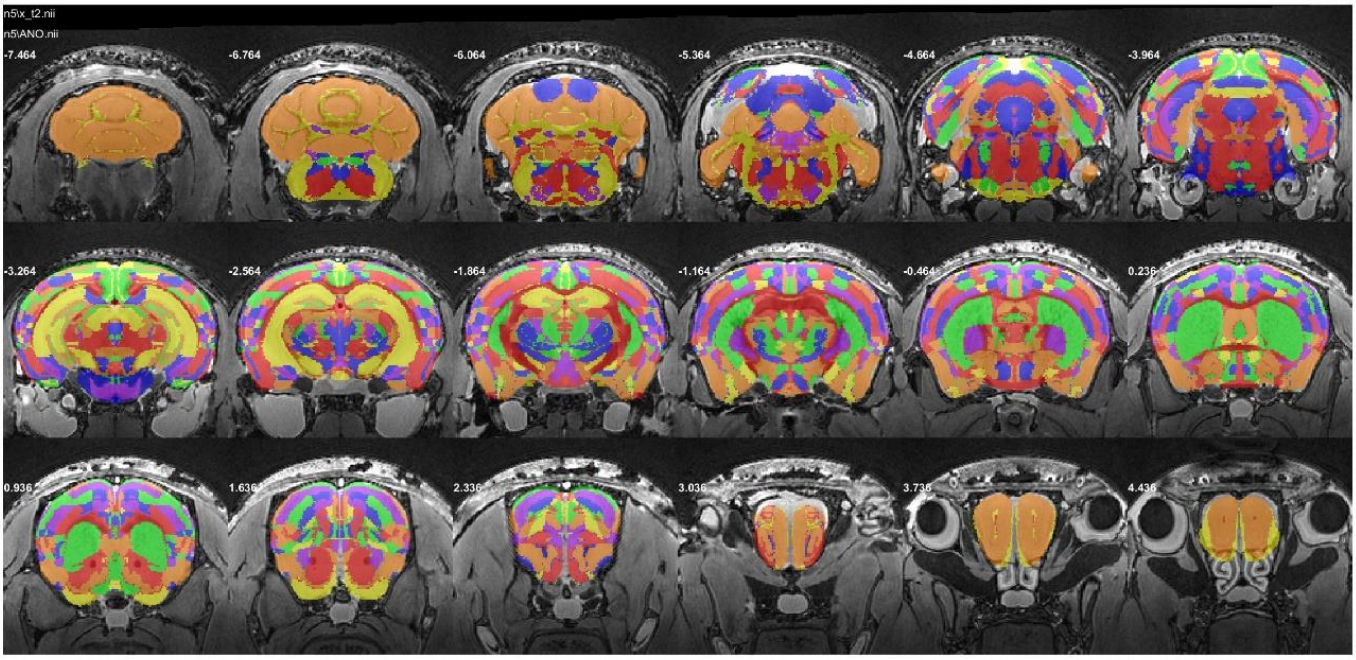
n5\x_t2.nii
n5\ANO.nii
-7.464
-6.764
-6.064
-5.364
-4.664
-3.964
-3.264
-2.564
-1.864
-1.164
-0.464
0.236
0.936
1.636
2.336
3.036
3.736
4.436

PTCH+/－_1

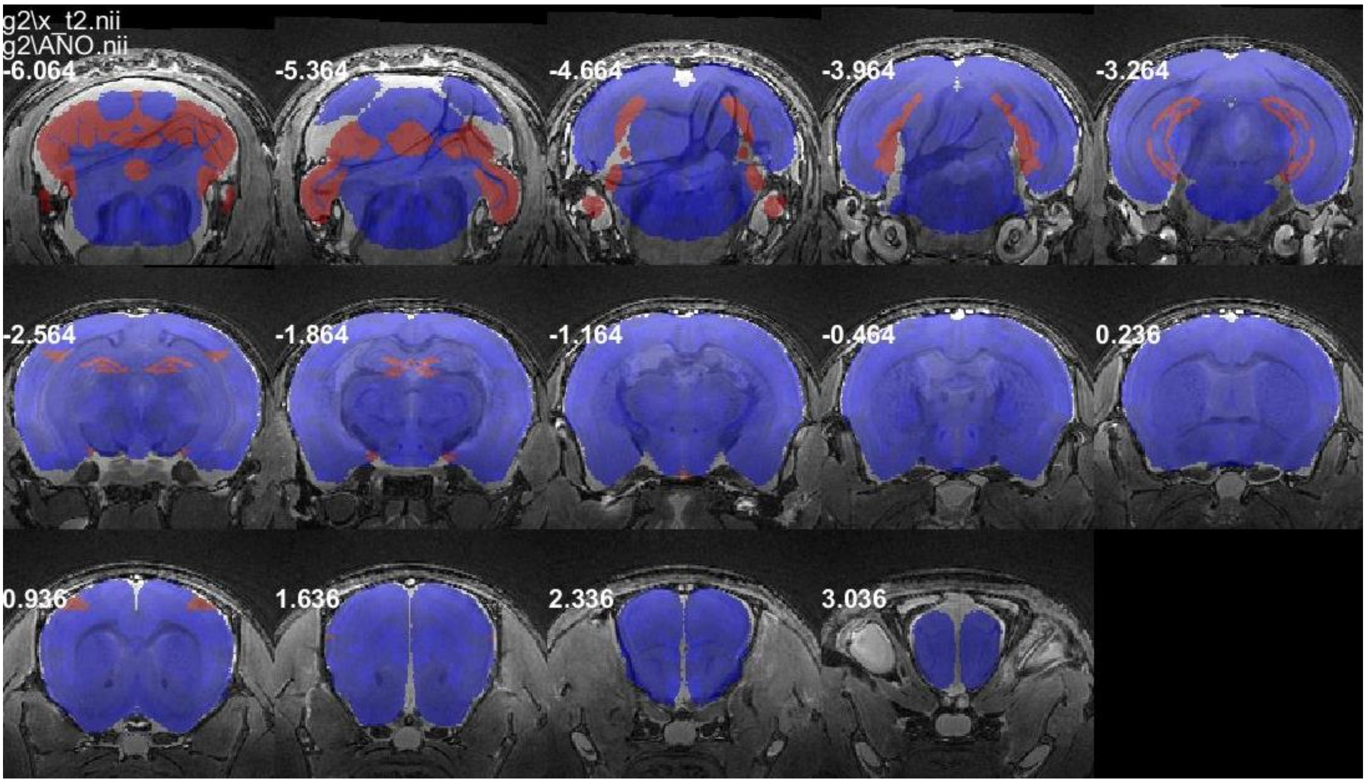
g2\x_t2.nii
g2\ANO.nii
-6.064
-5.364
-4.664
-3.964
-3.264
-2.564
-1.864
-1.164
-0.464
0.236
0.936
1.636
2.336
3.036

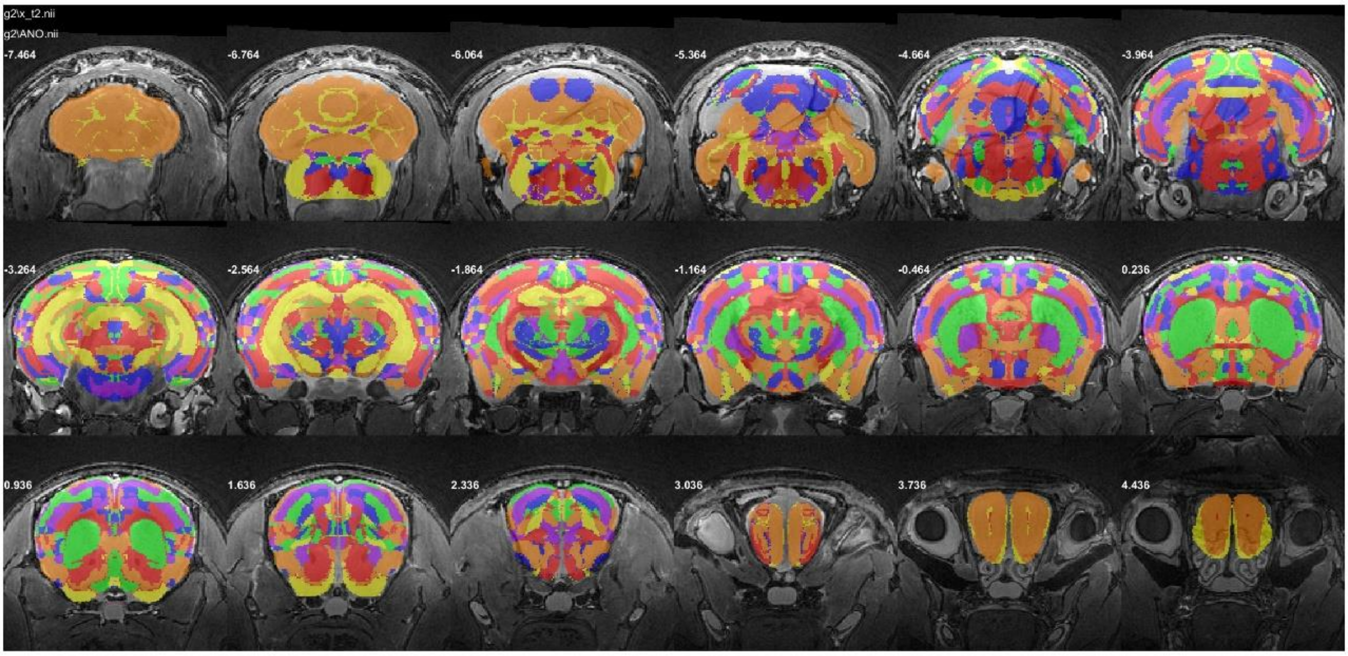
g2\x_t2.nii
g2\ANO.nii
-7.464
-6.764
-6.064
-5.364
-4.664
-3.964
-3.264
-2.564
-1.864
-1.164
-0.464
0.236
0.936
1.636
2.336
3.036
3.736
4.436

PTCH+/－_2

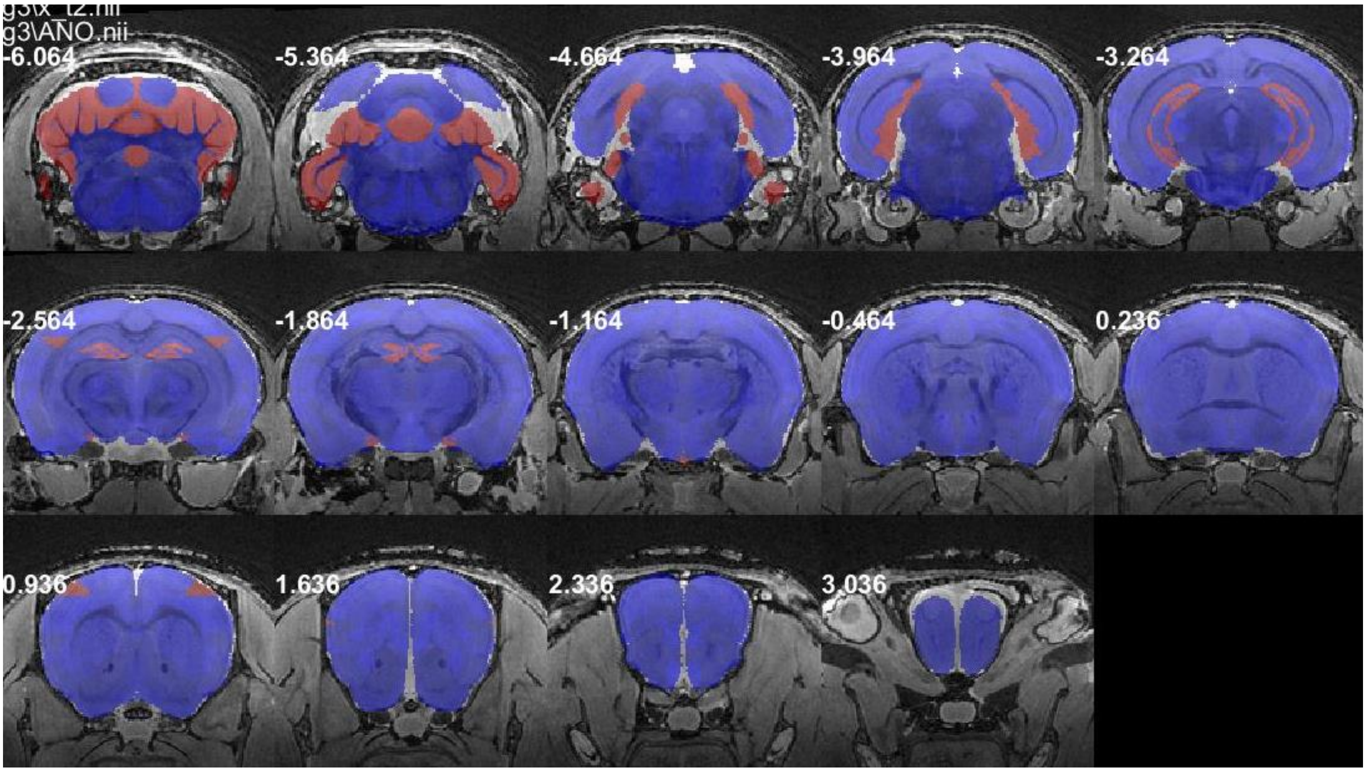
g3\x_t2.nii
g3\ANO.nii
-6.064
-5.364
-4.664
-3.964
-3.264
-2.564
-1.864
-1.164
-0.464
0.236
0.936
1.636
2.336
3.036

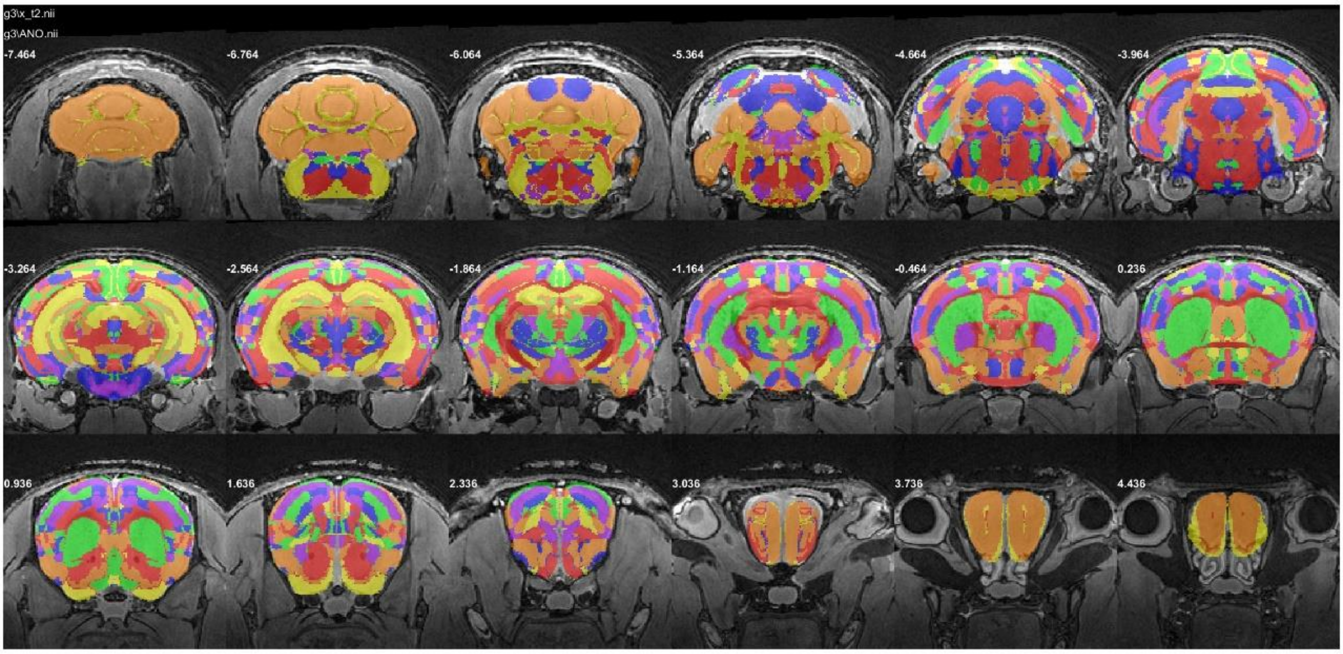
g3\x_t2.nii
g3\ANO.nii
-7.464
-6.764
-6.064
-5.364
-4.664
-3.964
-3.264
-2.564
-1.864
-1.164
-0.464
0.236
0.936
1.636
2.336
3.036
3.736
4.436

PTCH+/－_3

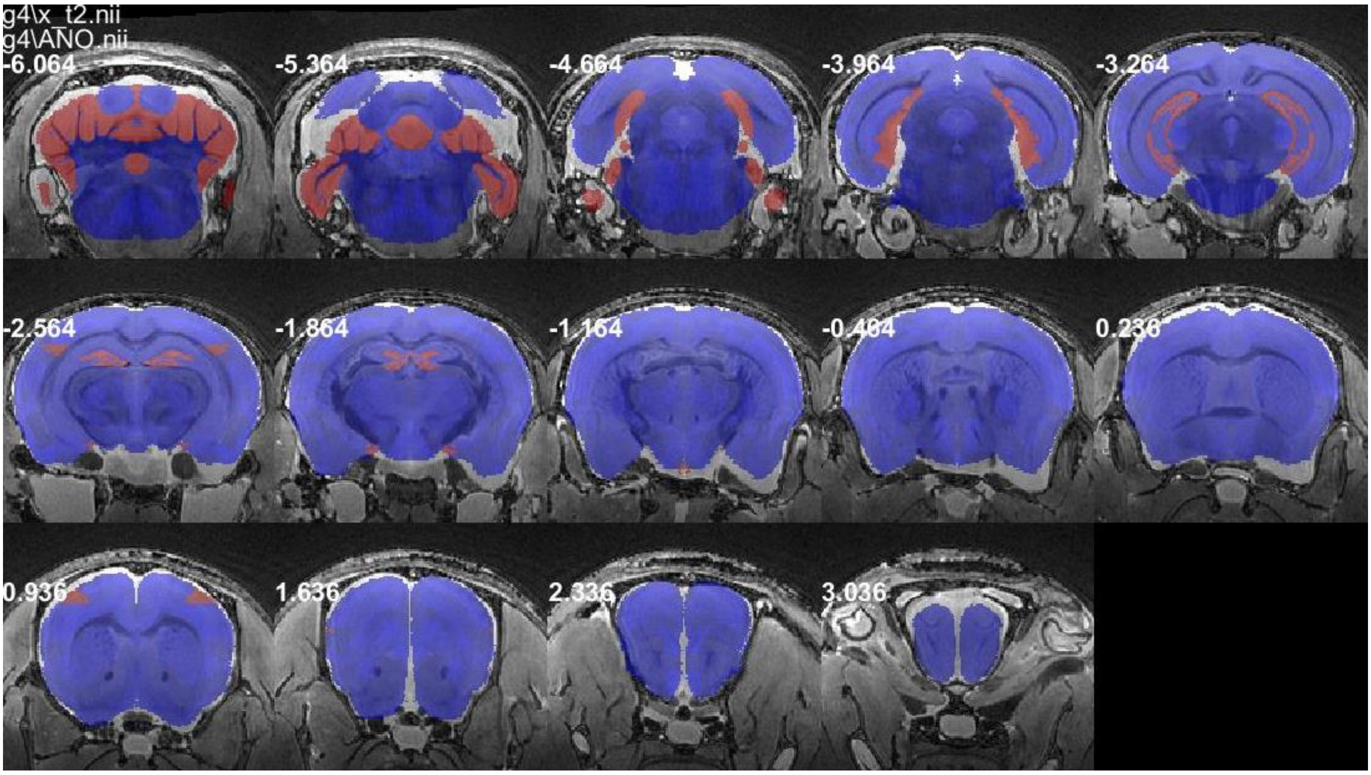
g4\x_t2.nii
g4\ANO.nii
-6.064
-5.364
-4.664
-3.964
-3.264
-2.564
-1.864
-1.164
-0.464
0.236
0.936
1.636
2.336
3.036

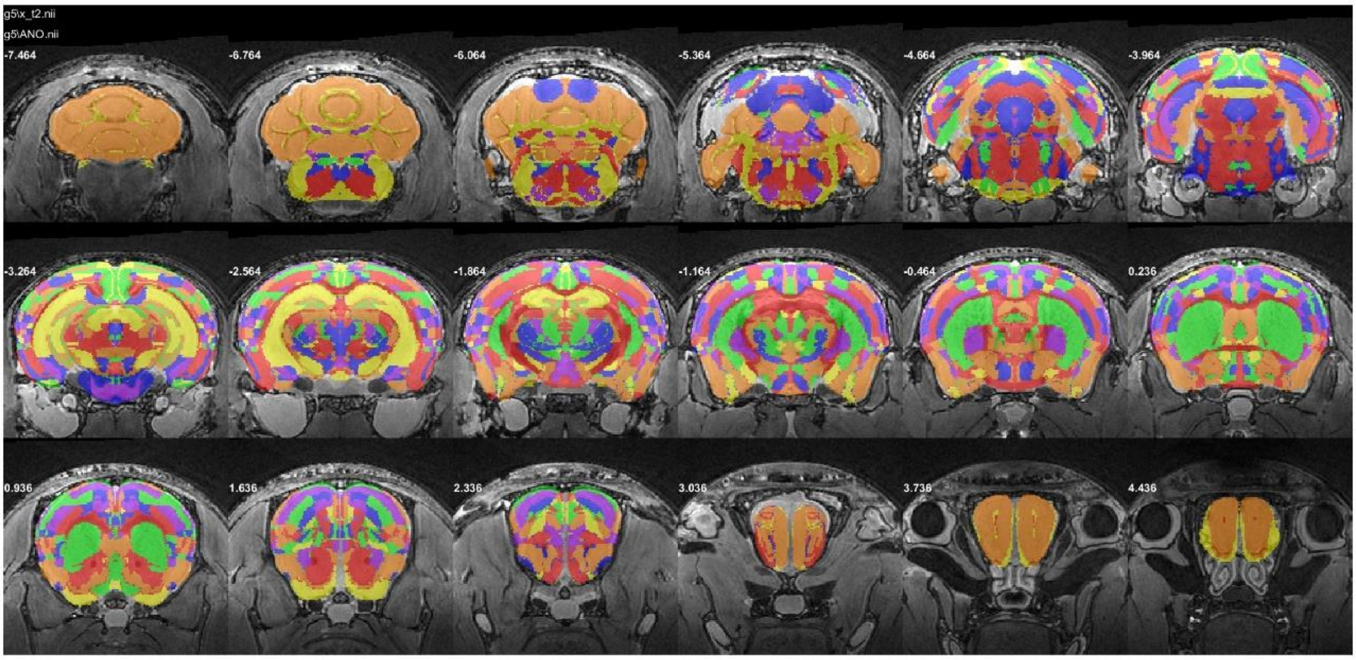
g5\x_t2.nii
g5\ANO.nii
-7.464
-6.764
-6.064
-5.364
-4.664
-3.964
-3.264
-2.564
-1.864
-1.164
-0.464
0.236
0.936
1.636
2.336
3.036
3.736
4.436

PTCH+/－_4

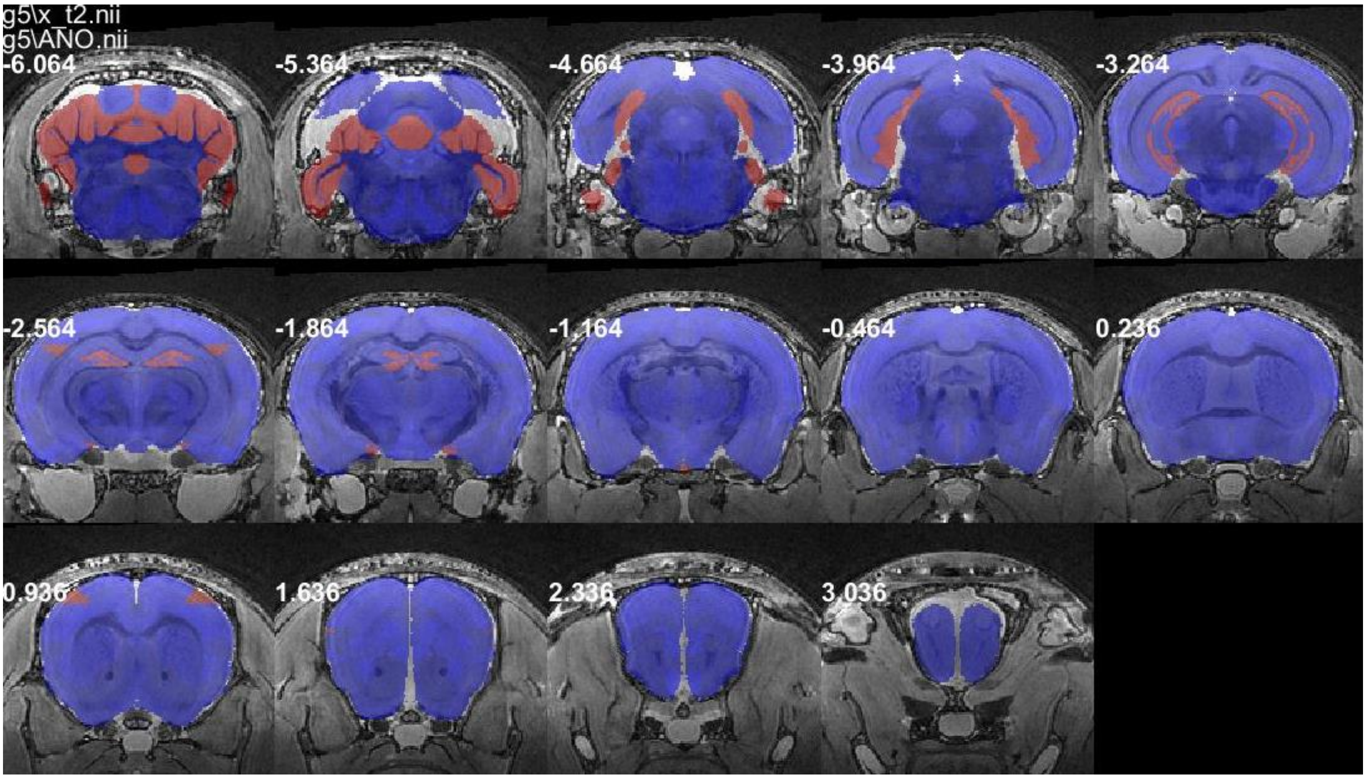
g5\x_t2.nii
g5\ANO.nii
-6.064
-5.364
-4.664
-3.964
-3.264
-2.564
-1.864
-1.164
-0.464
0.236
0.936
1.636
2.336
3.036

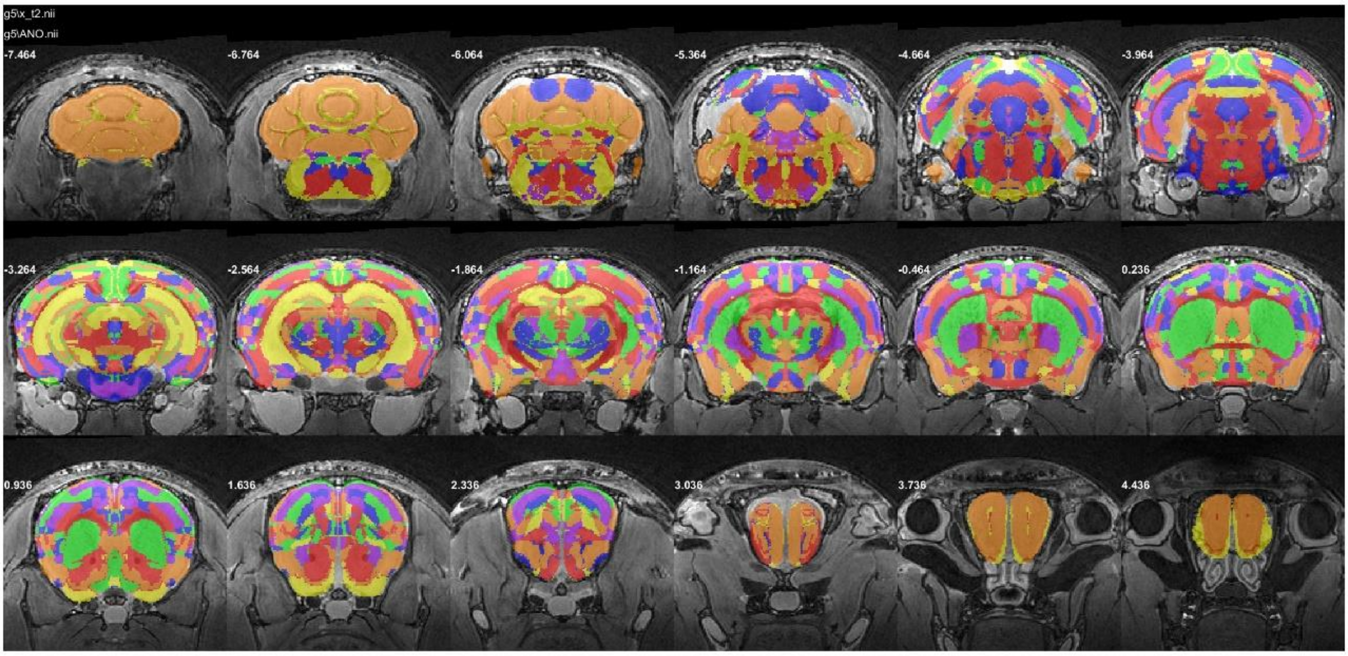
g5\x_t2.nii
g5\ANO.nii
-7.464
-6.764
-6.064
-5.364
-4.664
-3.964
-3.264
-2.564
-1.864
-1.164
-0.464
0.236
0.936
1.636
2.336
3.036
3.736
4.436

PTCH+/－_5

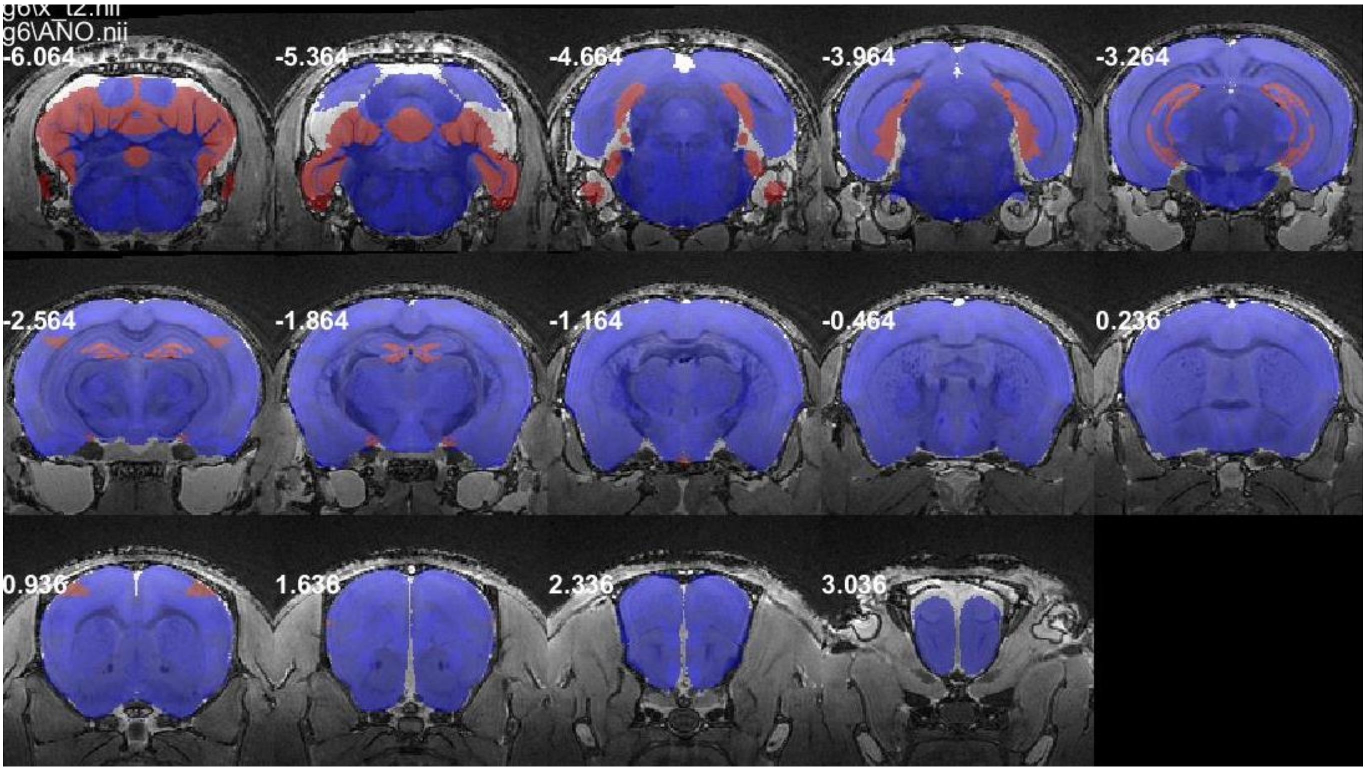
g6\x_t2.nii
g6\ANO.nii
-6.064
-5.364
-4.664
-3.964
-3.264
-2.564
-1.864
-1.164
-0.464
0.236
0.936
1.636
2.336
3.036

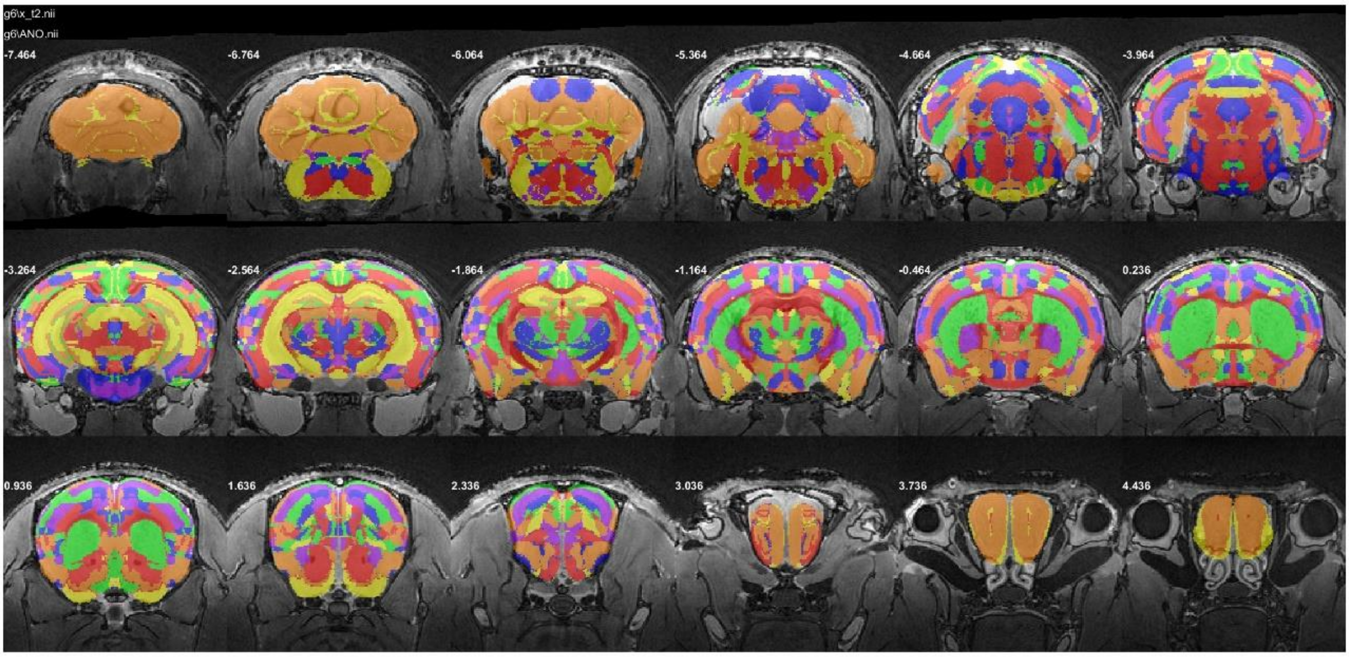
g6\x_t2.nii
g6\ANO.nii
-7.464
-6.764
-6.064
-5.364
-4.664
-3.964
-3.264
-2.564
-1.864
-1.164
-0.464
0.236
0.936
1.636
2.336
3.036
3.736
4.436
